# Reactive Oxygen Species‐Associated Chiral Nanoarchitectures for Bioscience

**DOI:** 10.1002/smsc.202300123

**Published:** 2023-11-27

**Authors:** Guangbo Yu, Aihua Qu, Zhimeng Wu, Liguang Xu, Chuanlai Xu, Hua Kuang

**Affiliations:** ^1^ International Joint Research Laboratory for Biointerface and Biodetection State Key Lab of Food Science and Technology School of Food Science and Technology Wuxi Jiangsu 214122 PRC; ^2^ The Key Laboratory of Carbohydrate Chemistry & Biotechnology Ministry of Education School of Biotechnology Jiangnan University Wuxi Jiangsu 214122 PRC

**Keywords:** biosensing, chiral nanoassemblies, chiral nanomaterials, reactive oxygen species regulation, tumor therapies

## Abstract

Chirality is a common occurrence in nature and forms a critical basis for life. The unique optical activity of chiral nanomaterials plays an important role in the nanobiology field, particularly in the regulation of reactive oxygen species (ROS), both in cells and in vivo. By regulating ROS, chiral nanomaterials can achieve numerous biological effects. Herein, progression in the application of chiral nanomaterials in the field of bioscience is introduced. First, the strategies used to construct different types of ROS‐related chiral nanomaterials are introduced. Then, the biological effects that can be achieved using chiral nanomaterials to regulate ROS are reviewed. Finally, the key issues and future prospects of chiral nanomaterials in the regulation of ROS are discussed.

## Graphical abstract

This review illustrates the strategies for constructing different types of ROS‐related chiral nanomaterials. The biological effects, the key issues, and future perspectives in the regulation of ROS are discussed.

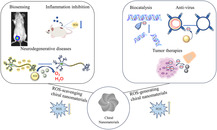

## 1 Introduction

Chirality exists in nature on all scales and its precise manifestation is that an object cannot be superimposed onto its mirror image. Chiral molecules can cause rotation of linearly polarized light; this can be characterized by circular dichroism spectra.^[^ ^1^ ^]^ Chiral molecules can be divided into left enantiomers and right enantiomers depending on the direction of rotation in linearly polarized light. Furthermore, chirality is closely related to the maintenance of normal physiological activities in organisms because there are many natural chiral molecules that play key biological roles, such as DNA, amino acids, and saccharides.^[^ ^2^ ^]^ The chirality of biomolecules exerts a selective effect on the molecules they bind to, thus influencing key reactions in biological systems.^[^ ^3^ ^]^ Although little is known about the origin of chiral properties, chirality has been studied extensively at the molecular level. Chiral molecules have similar physical properties but exhibit distinct biological effects. The development of nanotechnology has introduced the research and utilization of chirality from the molecular scale to the nanoscale; furthermore, the construction of chiral nanomaterials is a significant hotspot in current research.^[^ ^4^ ^]^ Compared with traditional nanomaterials, chiral nanomaterials possess greater advantages in terms of optical activity, biocompatibility, and safety; these properties promote wider application prospects in Raman detection,^[^ ^5^ ^]^ biosensing,^[^ ^6^ ^]^ microbial inhibition,^[^ ^7^ ^]^ radiotherapy,^[^ ^8^ ^]^ disease diagnosis,^[^ ^9^ ^]^ and therapy.^[^ ^10^ ^]^

Reactive oxygen species (ROS) is a general term for superoxide anions (O_2_·^−^), hydroxyl radicals (·OH), hydrogen peroxide (H_2_O_2_), and singlet oxygen (^1^O2).^[^ ^11^ ^]^ ROS can play a double role that is closely related to the physiological balance of homeostasis. At normal concentrations, ROS can act as a second messenger and play a role in signal transfer.^[^ ^12^ ^]^ Increasing ROS content within a cell's homeostatic environment can be beneficial to the organism.^[^ ^13^ ^]^ Studies have found that excessive ROS reshaped the early microbiota of *Drosophila melanogaster* and prolonged its lifespan.^[^ ^14^ ^]^ Similarly, in plants, an appropriate increase in ROS levels can trigger stress‐specific adaptation and defense mechanisms.^[^ ^15^ ^]^ However, once a specific threshold is exceeded, ROS will react nonspecifically with biological macromolecules, such as proteins and nucleic acids, thus disrupting homeostasis and causing oxidative stress in the physiological environment.^[^ ^13^ ^]^

To counter the cellular oxidative stress and damage caused by excess ROS, an organism usually maintains a balance between the production and removal of endogenous ROS via antioxidant enzymes and other antioxidant defense systems. Common antioxidant enzymes include superoxide dismutase,^[^ ^16^ ^]^ catalase,^[^ ^17^ ^]^ and glutathione reductase.^[^ ^18^ ^]^ Long‐term oxidative stress is implicated in many diseases, including cancer,^[^ ^19^ ^]^ neurodegenerative diseases, and inflammation.^[^ ^20^ ^]^ Therefore, the accurate monitoring and regulation of ROS levels are of great significance for understanding their biological role and coping with oxidative stress.

**Scheme 1:**
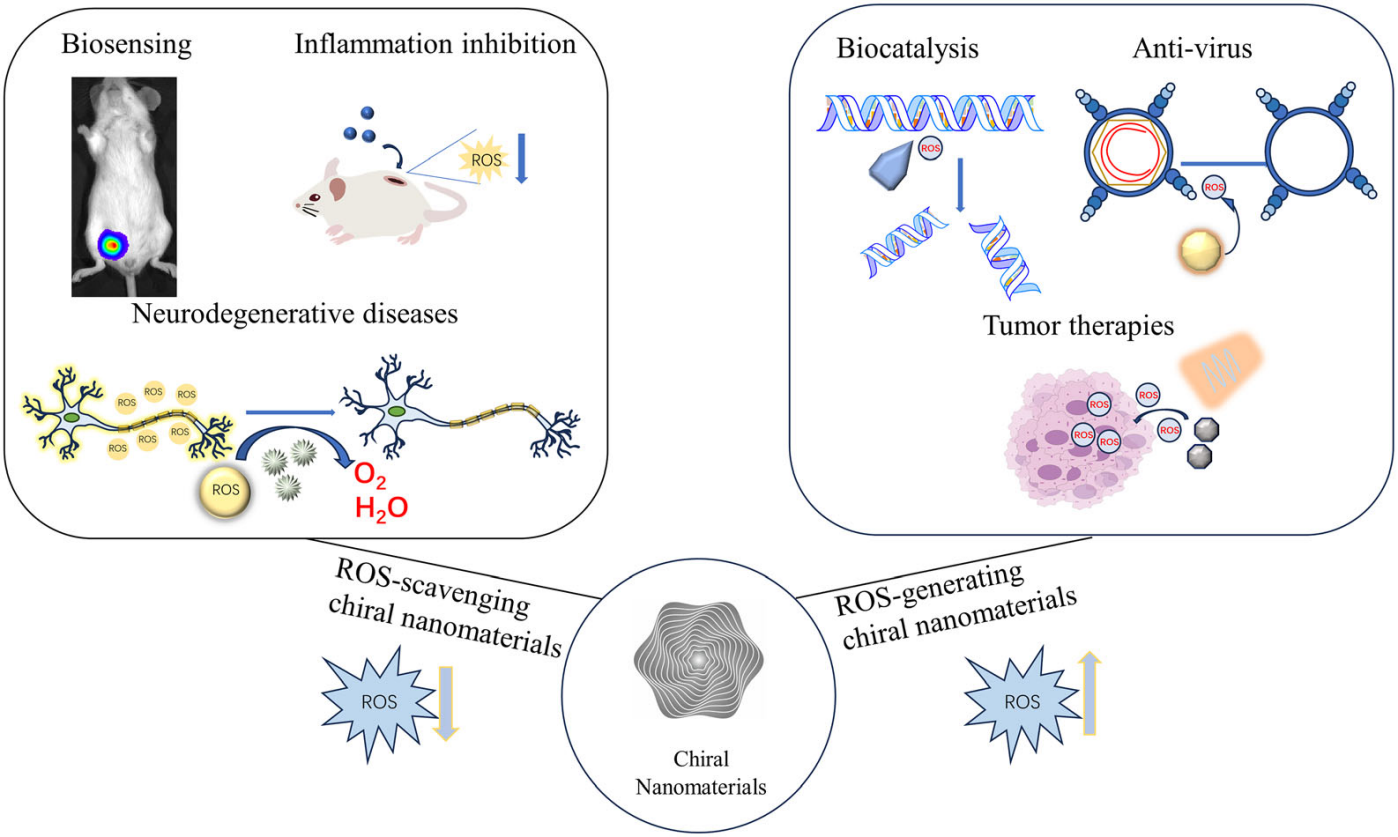
Chiral nanomaterials achieve biological applications by regulating ROS.

Traditional detection techniques, such as electron spin resonance (ESR), may not be sufficient to reflect the real‐time quantitative dynamics of ROS. Similarly, common biochemical analysis methods, such as small‐molecule fluorescent probes, are nonspecific and may be interfered by other types of ROS in the reaction system.^[^ ^21^ ^]^ In contrast, using nanomaterials for ROS monitoring and regulation offers specific advantages, including real‐time monitoring, high sensitivity, and high selectivity, thus extending ROS detection from cells to living organisms.^[^ ^22^ ^]^ When constructing nanomaterials that regulate ROS, it is crucial to consider the system's nanobiotic interactions, as nanomaterial–biomolecular interactions can influence cell uptake and even alter the function of nanomaterials.^[^ ^23^ ^]^ Some studies have demonstrated that the chirality of nanomaterials significantly influenced the interaction between biological systems and nanomaterials. For instance, Nie found that the energy metabolism of T24 cells exhibited a strong chiral dependence.^[^ ^24^ ^]^ Furthermore, chiral mesostructured hydroxyapatite films (CMHAPFs) exhibit enantioselectivity in the regulation of cell proliferation and the osteogenic differentiation of primary mesenchymal stem cells.^[^ ^25^ ^]^ The modification of nanomaterials with chiral ligands reduces their cytotoxicity, thus indicating better biocompatibility.^[^ ^26^ ^]^ Furthermore, chiral nanomaterials can enhance cell uptake and regulate cell behavior, making them an advantageous strategy for monitoring ROS.^[^ ^27^ ^]^ Therefore, the use of chiral nanomaterials to monitor ROS represents a highly promising strategy. Chiral nanomaterials can achieve biological effects by regulating ROS content and represent significant promise for application in biosensing, biocatalysis, microbial inhibition, disease diagnosis, and treatment (**Scheme** **1**). Overall, chiral nanomaterials hold great potential for advancing the field of ROS research and its applications in various biomedical areas.

The purpose of this review was to present the current research status of chiral nanomaterials for the regulation of ROS. First, we outlined the construction methods and sources of chirality for ROS‐related chiral nanomaterials, focusing on two main aspects: individual chiral nanoparticles and chiral nanoassemblies. Next, we provided an overview of the specific application of chiral nanomaterials in monitoring and regulating ROS in cells or in vivo, including biosensing and tumor therapy. Finally, we discussed the limitations and future prospects of chiral nanomaterials in the regulation of ROS.

## 2 Construction of ROS‐Associated Chiral Nanomaterials

The synthesis of nanomaterials can generally be classified into two approaches: top‐down and bottom‐up.^[^ ^28^ ^]^ Of these, the bottom‐up method has emerged as the primary research direction for investigating chiral nanomaterials and has yielded remarkable results.^[^ ^29^ ^]^ The bottom‐up method relies on precise synthetic strategies and self‐assembly principles to gradually construct chiral structures at the nanoscale and precisely regulate their properties and functions.^[^ ^30^ ^]^ This approach utilizes the interaction between chiral molecules and the self‐assembly behavior of nanomaterials to precisely control the synthesis of chiral nanomaterials by designing and controlling factors such as chiral ligands, chiral inducers, or chiral templates. The chirality of inorganic nanomaterials can generally be attributed to three sources: 1) nanostructures with asymmetric chiral morphology^[^ ^31^ ^]^; 2) the modification of achiral nanomaterials with chiral ligands^[^ ^32^ ^]^; and 3) chiral templates that assemble with achiral nanomaterials to form chiral assemblies.^[^ ^33^ ^]^ Chiral nanomaterials exhibit unique optical activity, as well as enhanced biocompatibility and stability compared to inorganic nanoparticles. The synthesis of chiral nanomaterials of various sizes and morphologies has achieved significant progress over the past decade, particularly with the advancements in bioinspired chiral inorganic nanomaterials.

**Figure 1:**
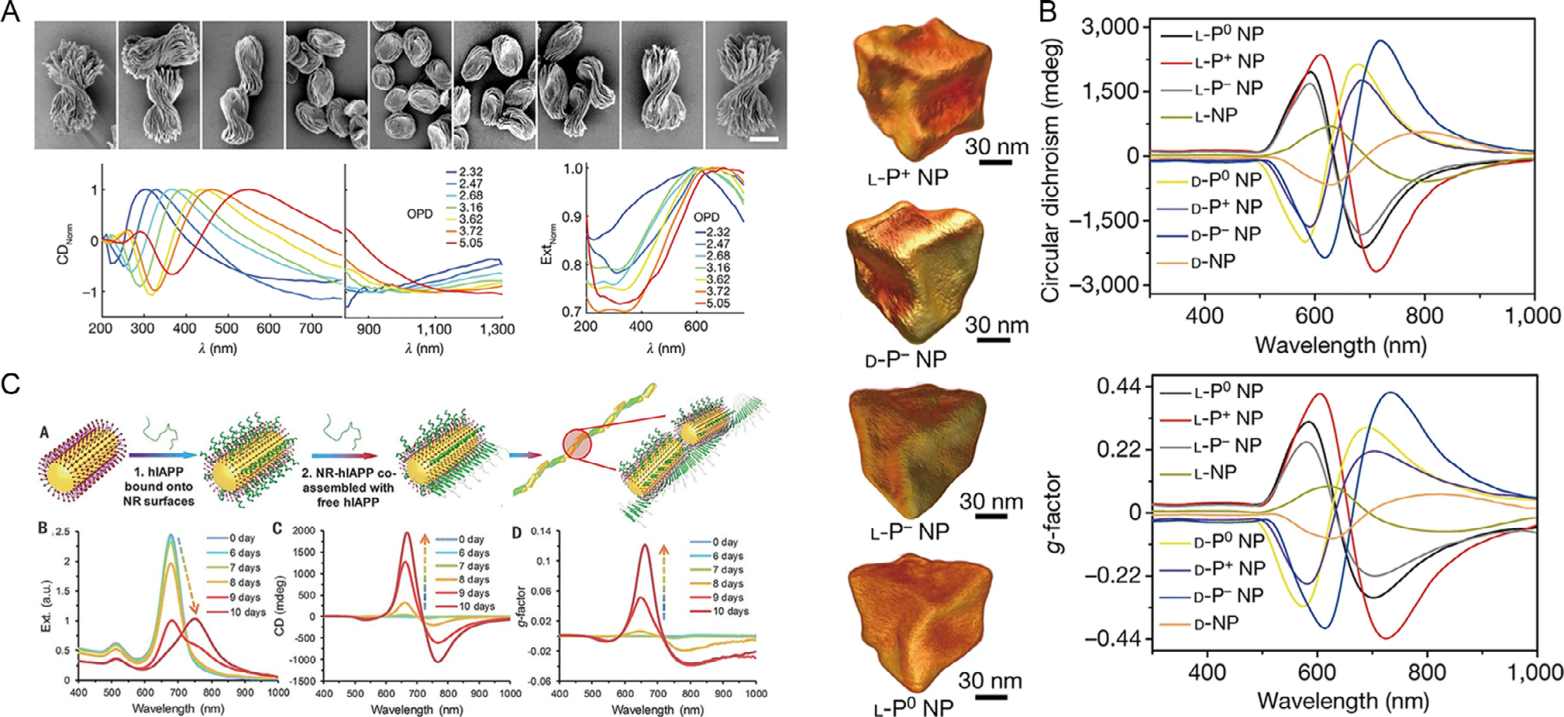
A) SEM images of the bowtie particles from left‐handed to pancake to right‐handed and spectroscopy of chiral continuity. Reproduced with permission.^[^ ^31^ ^]^ Copyright 2023, Springer Nature. B) Transmission electron microscope (TEM) images and spectroscopy of photosynthesized chiral NPs. Reproduced with permission.^[^ ^34^ ^]^ Copyright 2022, Springer Nature. C) Schematic diagram of gold nanorods promoting fibrosis and coassembly of hIAPP and spectroscopy of the assembly. Reproduced with permission.^[^ ^35^ ^]^ Copyright 2021, American Association for the Advancement of Science.

Kotov et al. conducted a study in which Cd^2+^ solution was mixed with cystine to obtain bowtie‐shaped chiral nanostructures.^[^ ^31^ ^]^ The circular dichroism peaks of these structures were attributed to absorption and scattering phenomena (**Figure** **1**A). Unlike traditional chiral molecules, circular dichroism measurements revealed that the chirality of the nanostructure was continuous. Moreover, by adjusting various reaction conditions, it was possible to produce bowtie‐shaped chiral nanostructures with different morphologies. In another study, Xu et al. induced the growth of gold nanoparticles using chiral ligands L‐or D‐cysteine‐phenylalanine dipeptides and introduced polarized light during the preparation process.^[^ ^34^ ^]^ The application of polarized light broke the symmetry on the high‐index crystal surface, and the chirality of the photons was transferred to the nanoparticles (Figure 1B). As a result, chiral gold nanoparticles with uniform surface morphology and an anisotropy factor of 0.44 were obtained. Liu et al. used supramolecular interactions to assemble gold nanorods and human amylin, thus resulting in the construction of helical fibrous chiral nanoassemblies that were similar to chiral liquid crystals.^[^ ^35^ ^]^ Notably, when the size of the nanorods matched the geometry of the human amylin helix, the g‐factor was as high as 0.12 during the assembly process (Figure 1C). These studies have significantly advanced our understanding of the fundamental principles involved in constructing chiral nanomaterials with special surface properties and optical activities. Research findings have provided valuable insights and assistance for the preparation of chiral nanomaterials. In this section, we introduced representative construction methods for ROS‐related chiral nanomaterials and their chiral sources, which predominantly included the preparation of single‐chiral nanoparticles using chiral molecules as ligands and the assembly of inorganic nanoparticles through templates.

## 3 Individual Chiral NPs

**Figure 2:**
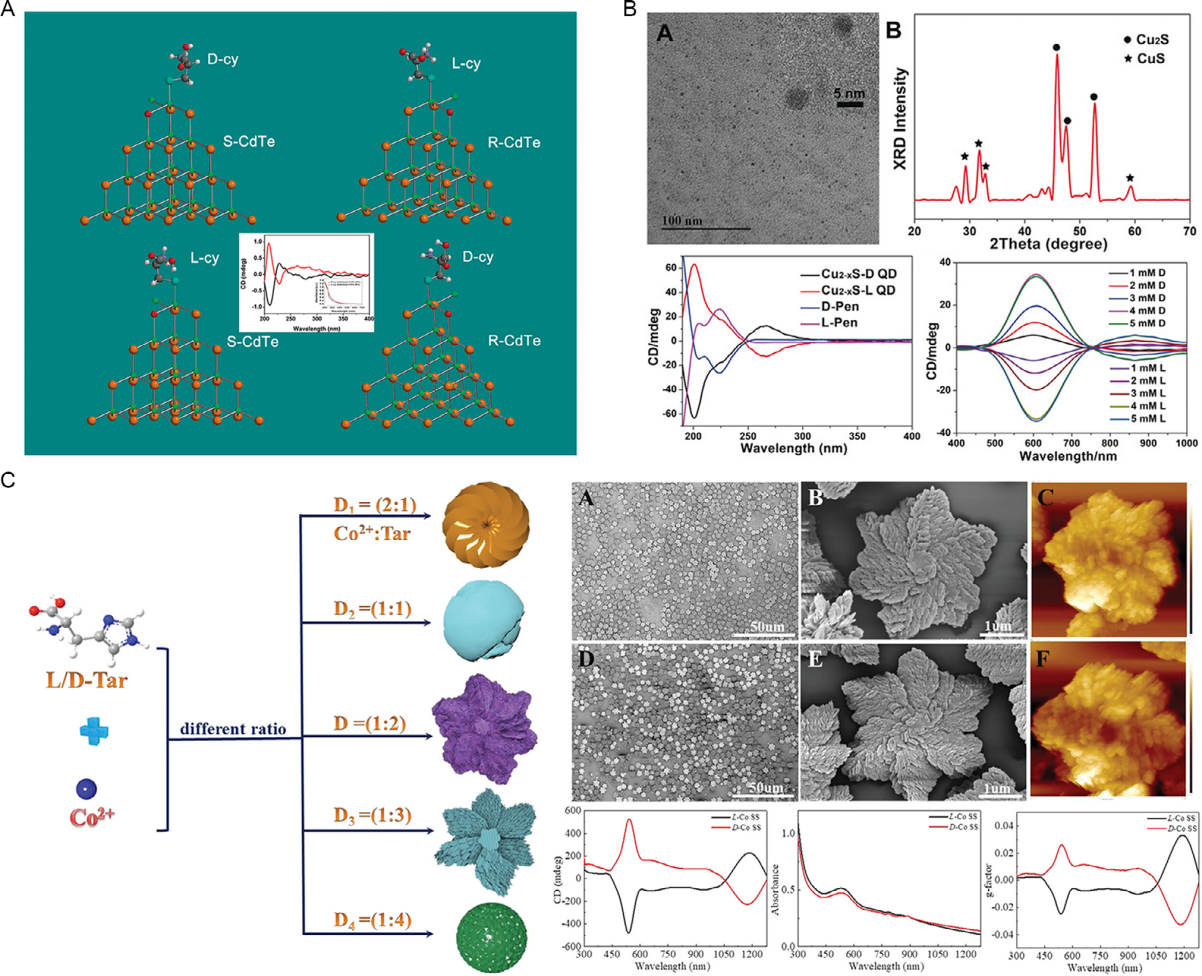
A) Spectra of cysteine‐stabilized CdTe NCs and corresponding models. Reproduced with permission.^[^ ^36^ ^]^ Copyright 2010, American Chemical Society. B) TEM images, X‐ray diffraction (XRD), and spectroscopy of L‐QDs. Reproduced with permission.^[^ ^37^ ^]^ Copyright 2019, Wiley‐VCH. C) Schematic diagram of chiral Co SS and SEM images and spectra of hexagonal star morphology. Reproduced with permission.^[^ ^38^ ^]^ Copyright 2022, Wiley‐VCH.

Small size and surface chemistry enable chiral quantum dots (QDs) to be used in photocatalysis. Kotov reported the effect of cysteine on the optical isomerism, growth rate, and structure of chiral CdTe QDs.^[^ ^36^ ^]^ Cysteine served as a stabilizer to induce chirality on the core of CdTe nanocrystals. By investigating CdTe nanocrystal growth kinetics, it was observed that D‐Cysteine exhibited better interaction with CdTe nanocrystals when compared to L‐Cys. Calculations and experimental data further revealed that the atomic arrangement of chiral cysteine‐stabilized CdTe nanocrystals was tetrahedral and that chirality emerged when all vertices of the tetrahedrons were different, similar to an organic compound (**Figure** **2**A). In another study, Hao et al. constructed chiral copper sulfide QDs (Cu_2−*x*_S QDs) using penicillamine as a ligand; the anisotropy factor of the Cu_2−*x*_S QDs reached 0.01, possibly due to the strong coordination bonds between copper and sulfur atoms.^[^ ^37^ ^]^ Transmission electron microscopy further showed that the core of Cu_2−‐*x*_ S QDs was achiral (Figure 2B). In addition, the chiral activity caused by dipolar interactions was weak due to the small dielectric constant. Hence, the most likely cause of chirality in Cu_2−*x*_S QDs was attributed to the asymmetric electrostatic field. Both QDs could generate hydroxyl radicals under the irradiation of circularly polarized light. Wang et al. adjusted the concentration of chiral ligands to obtain chiral cobalt nanostructures with different morphologies^[^ ^38^ ^]^ and showed that L/D‐Tar and Co^2+^ combined to induce the formation of a superstructure of Co^2+^ (Figure 2C). The coordination bond between L/D‐Tar and Co^2+^ influenced the molecular mechanism of the chiral structure, thus resulting in the chiral signal shifting from the ultraviolet region to the infrared region.

## 4 Chiral Nanoassemblies

**Figure 3:**
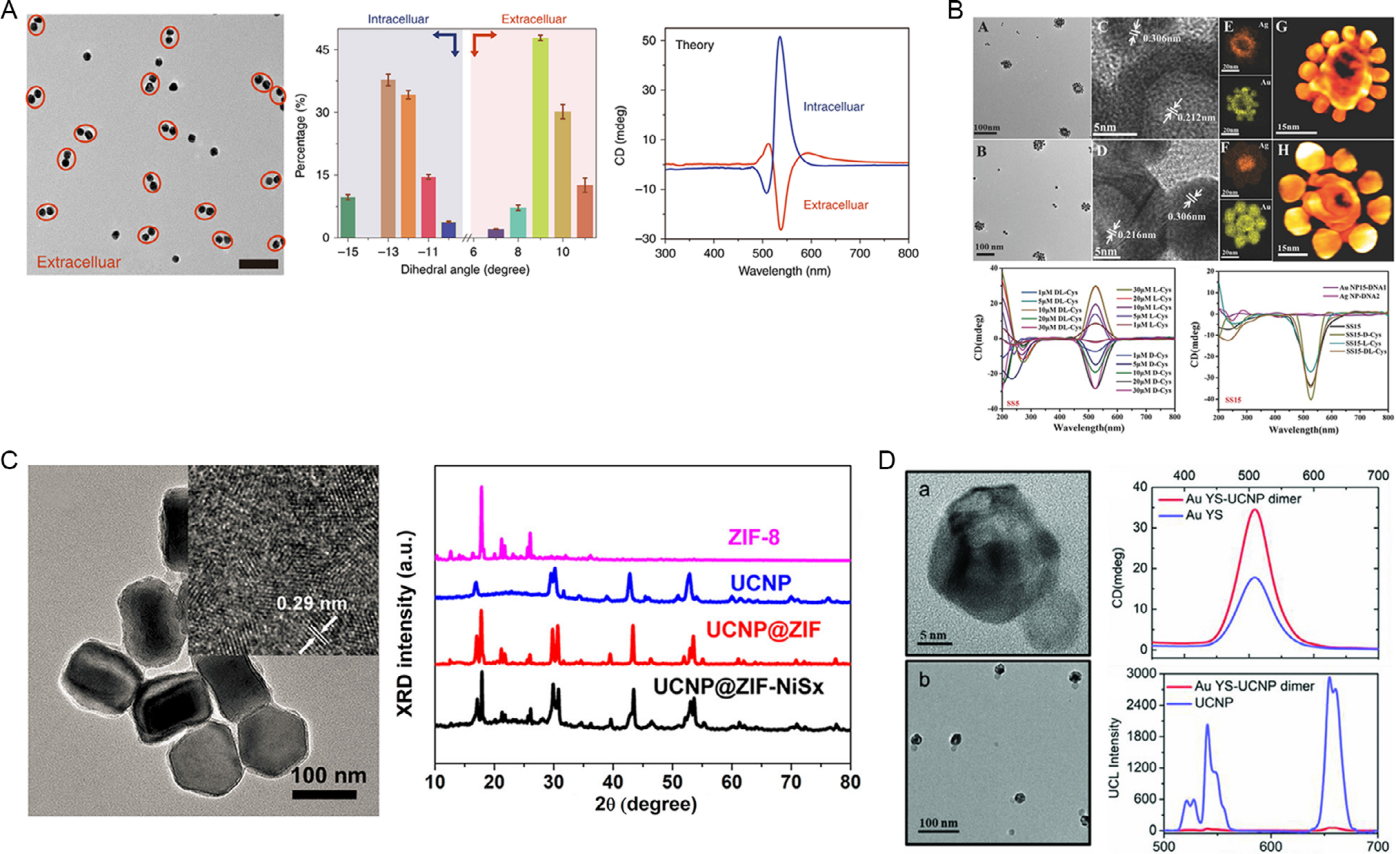
A) TEM image and simulated spectra of NP dimers based on geometries. Reproduced with permission.^[^ ^45^ ^]^ Copyright2018, Springer Nature. B) TEM images and spectra of SS nanostructures. Reproduced with permission.^[^ ^46^ ^]^ Copyright2017, Wiley‐VCH. C) TEM image and XRD spectra of UCNP@ZIF‐NiS_*x*_ nanoassemblies. Reproduced with permission.^[^ ^47^ ^]^ Copyright 2019, American Chemical Society. D) TEM image and CD spectra of Au YS–UCNP heterodimer. Reproduced with permission.^[^ ^48^ ^]^ Copyright 2018, Wiley‐VCH.

The construction of chiral nanomaterials through the self‐assembly of nanoparticles using templates is another prominent approach.^[^ ^39^ ^]^ Chiral nanocomponents have been extensively manufactured over recent years and have demonstrated good stereo conformation and tunable plasmon‐enhanced chiral activity; these properties are advantageous for enhancing drug utilization efficiency and reducing side effects.^[^ ^40^ ^]^ Multifunctional templates adsorb inorganic nanoparticles to their surfaces through covalent or noncovalent interactions to form components with certain spatial geometric structures, including dimers,^[^ ^41^ ^]^ trimers,^[^ ^42^ ^]^ core satellites,^[^ ^43^ ^]^ and other structures. One effective template for regulating the arrangement of nanoparticles is DNA with its double‐helix structure offering designability and predictability.^[^ ^44^ ^]^ Sun constructed DNA‐bridged chiral dimeric Au nanoparticles (NP) via the self‐assembly of achiral Au NPs.^[^ ^45^ ^]^ The dimer exhibited a geometrically chiral morphology, thus resulting in the generation of circular dichroism signals (**Figure** **3**A). Interestingly, the chiral signal was reversed when the dimer was exposed to cell culture medium, indicating that changes in the environment can alter the chiral configuration of this material. This emphasizes the significance of considering nanobiological interactions when designing chiral nanomaterials. Another example involves the assembly of Ag nanoparticles as cores and Au nanoparticles as satellites, mediated by complementary DNA sequences, to form core–satellite nanostructures.^[^ ^46^ ^]^ The dihedral angle between the core and satellite NPs contributes to the CD signal of the core–satellite nanostructures which arises from an asymmetric dipole–dipole interaction between the nanoparticles (Figure 3B). Upon modification with L‐/D‐Cys, the CD signal of the nanocomponent was significantly enhanced, thus indicating the influence of chiral molecules as another reason for CD signal generation. The chiral shell–satellite structure (SS) functions as a potent chiral photosensitizer, thus exhibiting a much higher ROS generation ability when compared to traditional photosensitizers.

Upconversion particles (UCNPs) are also frequently used as linker molecules. For instance, using L/D‐Pen as a chiral ligand, chiral NiS_*x*_ was successfully synthesized in UCNP‐ZIF8 after PVP‐coated UCNP was coated with a layer of ZIF‐8 to form a shell structure.^[^ ^47^ ^]^ The CD spectra of UCNP@ZIF‐NiS_*x*_‐L showed two strong CD signals, thus indicating that the chirality of the assemblies originated from chiral NiS_*x*_‐L NPs (Figure 3C). Polymyxin B‐modified UCNPs combined with polymyxin B antibody‐coated gold yellow‐shell (Au YS) NPs was also used to generate chiral dimers.^[^ ^48^ ^]^ The CD signal was enhanced after heterodimer assembly, and under 980 nm irradiation, the chiral dimer exhibited ROS generation for photodynamic therapy (Figure 3D). The enhancement of chirality includes two possible factors: the ellipsoidal shape of the NPs to form a chiral conformation after assembly^[^ ^49^ ^]^ and the plasma resonance caused by the shell structure.^[^ ^46^ ^]^ Under 980 nm irradiation, this chiral dimer can generate ROS for photodynamic therapy. The self‐assembly of nanoparticles using templates offers a powerful method with which to create chiral nanomaterials with unique properties; furthermore, these structures hold great promise for various applications in biomedicine and beyond.

## 5 ROS‐Associated Chiral Nanomaterials for Bioapplication

According to their specific function in ROS regulation, ROS‐related chiral nanomaterials can be categorized into two main types: ROS‐scavenging chiral nanomaterials and ROS‐generating chiral nanomaterials. ROS‐scavenging chiral nanomaterials act as exogenous antioxidants and are designed to scavenge excessive ROS when endogenous antioxidants are unable to fully clear them, thereby helping to maintain cellular homeostasis. ROS‐scavenging chiral nanomaterials can be applied in various aging‐related diseases, such as inflammation and neurodegenerative diseases. ROS‐generating chiral nanomaterials exploit the cytotoxic effects of ROS to induce damage at specific target sites. The most significant application of ROS‐generating chiral nanomaterials is in cancer therapy. These nanoparticles can be designed to selectively target tumor cells and generate a substantial amount of ROS to induce cell death and destroy tumors.

In this section, we comprehensively review research progress in the field of ROS‐related chiral nanomaterials, specifically focusing on their applications in various areas. First, chiral nanomaterials can be used as biosensors to detect and monitor ROS levels in biological systems, thus providing valuable information relating to oxidative stress and cellular health. Second, in biocatalysis, chiral nanomaterials can be employed as catalysts in ROS‐related biocatalytic reactions, thus facilitating specific chemical transformations and promoting ROS‐related processes. Third, in microbial inhibition, chiral nanomaterials may be used to inhibit the growth of harmful microorganisms through ROS‐mediated antimicrobial effects. Fourth, in disease diagnosis and treatment, chiral nanomaterials hold significant promise for the diagnosis and treatment of various diseases, such as cancer and other ROS‐related disorders, by exploiting their ROS‐scavenging or ROS‐generating properties. Here, we review research progress and the potential applications of ROS‐related chiral nanomaterials in these areas, providing insights into their utility and potential impact in various biomedical and environmental applications.

## 6 Biosensing and Bioimaging

ROS plays a key role in many physiological processes along with health and disease. Its deficiency or excess will cause physiological conditions to be out of balance and cause related diseases. Therefore, the rapid and accurate determination of ROS levels is crucial. Traditional detection methods such as fluorescent probes^[^ ^50^ ^]^ are limited by poor biocompatibility, low sensitivity, and photobleaching effects. Chiral nanomaterials can solve these problems.

**Figure 4:**
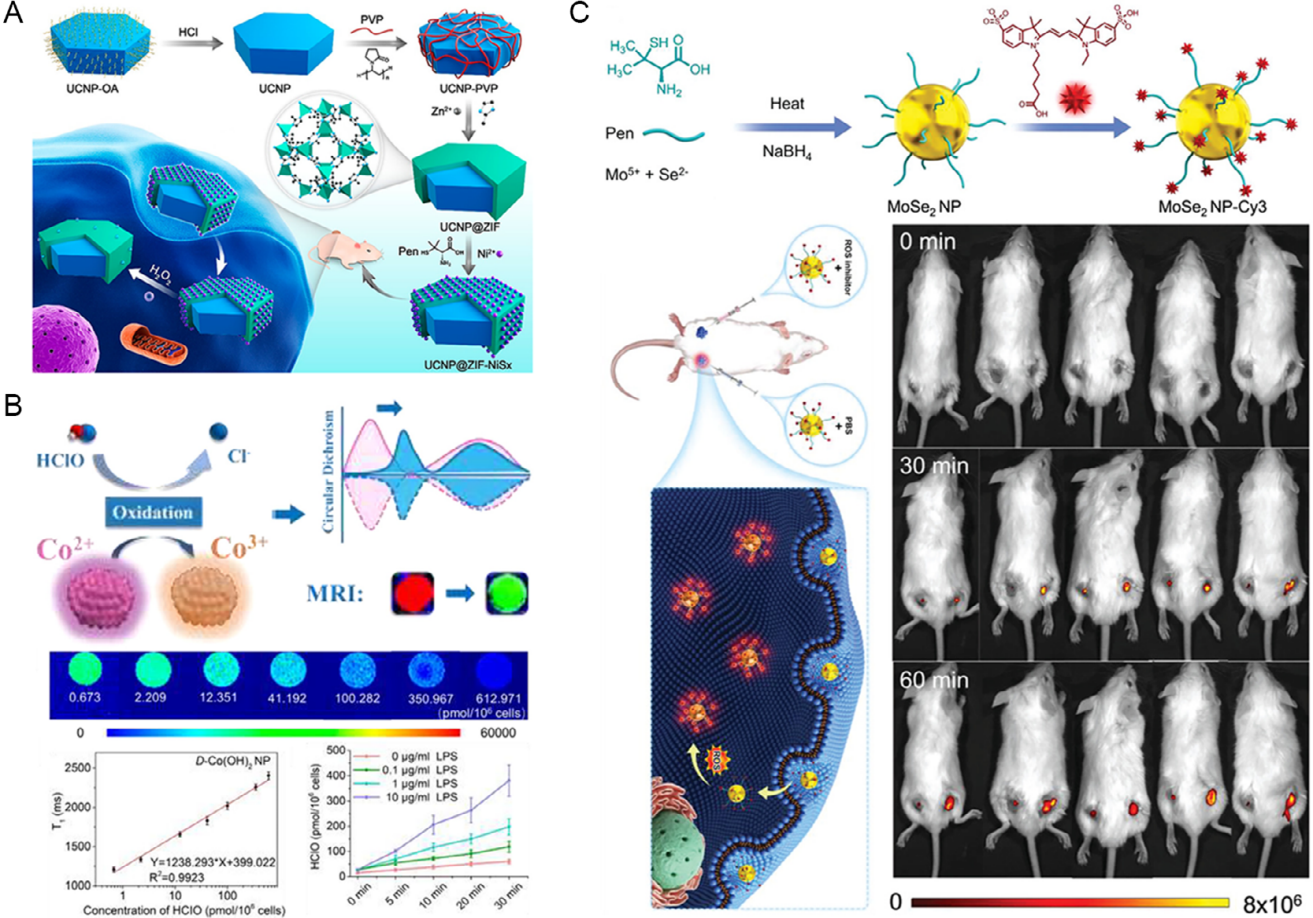
A) Chiral UCNP@MOF‐NiS_*x*_ nanoassemblies for UCL imaging of ROS both in vitro and in vivo. Reproduced with permission.^[^ ^47^ ^]^ Copyright 2019, American Chemical Society. B) Chiral Co(OH)_2_ NPs for ROS detection in tumor‐bearing mice through fluorescence and MRI dual imaging. Reproduced with permission.^[^ ^51^ ^]^ Copyright 2022, American Chemical Society. C) Chiral MoSe_2_ for fluorescence imaging of ROS both in vitro and in vivo. Reproduced with permission.^[^ ^52^ ^]^ Copyright 2023, Wiley‐VCH.

In a previous study, Hao prepared chiral nanoassemblies consisting of an UCNP core and a ZIF‐8 shell encapsulated by chiral NiS_*x*_ NPs.^[^ ^47^ ^]^ UCNP@ZIF‐NiS_*x*_ can be utilized for the quantitative monitoring of H_2_O_2_. The CD signal and upconversion luminescence (UCL) signal of these nanoassemblies can be employed as dual‐mode detection techniques (**Figure** **4**A). The release of chiral NiS_*x*_ due to the catalytic effect of H_2_O_2_ results in a reduction in the CD signal, while the recovery of upconversion luminescent signal indicates the transformation from UCNP@ZIF‐NiS_*x*_ to UCNP@ZIF, thus enabling the quantitative detection of H_2_O_2_. This strategy demonstrates that MOF acts as a biocompatible linker and can be applied to the synthesis of chiral nanoassemblies; in addition, the dual mode can also be used for the detection of other markers. Co(OH)_2_ nanoparticles with both chiral and magnetic properties have been used for the dual‐mode detection of hypochlorous acid in cells and in vivo.^[^ ^51^ ^]^ D‐aspartic acid‐modified Co(OH)_2_ nanoparticles showed better sensitivity and were able to detect intracellular ROS from 0.673 to 612.971 pmol/10^6^ cells (Figure 4B). In 4T1 model mice, d‐Co(OH)_2_ NPs enabled the dynamic monitoring of hypochlorous acid by both fluorescence and MRI signals.

In addition to nuclear magnetic imaging and UCL, the combination of chiral recognition and fluorescent molecules can also achieve dual‐signal detection. Chiral MoSe_2_ nanoparticles functionalized with penicillamine ligands have been coupled with Cy3 fluorescent molecules to create nanoprobes.^[^ ^52^ ^]^ The chrial nanoprobes exhibit prominent CD signals and effectively quench the fluorescence of Cy3 (Figure 4C). In the presence of H_2_O_2_, a redox reaction occurs, thus leading to a significant reduction in the CD signal and the simultaneous recovery of fluorescence signals. This phenomenon is attributed to the oxidation reaction of the nanoprobes induced by hydrogen peroxide, which alters their electronic structures, thus affecting the generation of CD signals and restoring the fluorescence emission. These examples demonstrate the potential of chiral nanomaterials for ROS detection using various detection modes. Chiral nanomaterials provide versatile tools for sensitive and specific ROS detection, offering promising applications in biomedical research and diagnostics.

## 7 Biocatalysis

**Figure 5:**
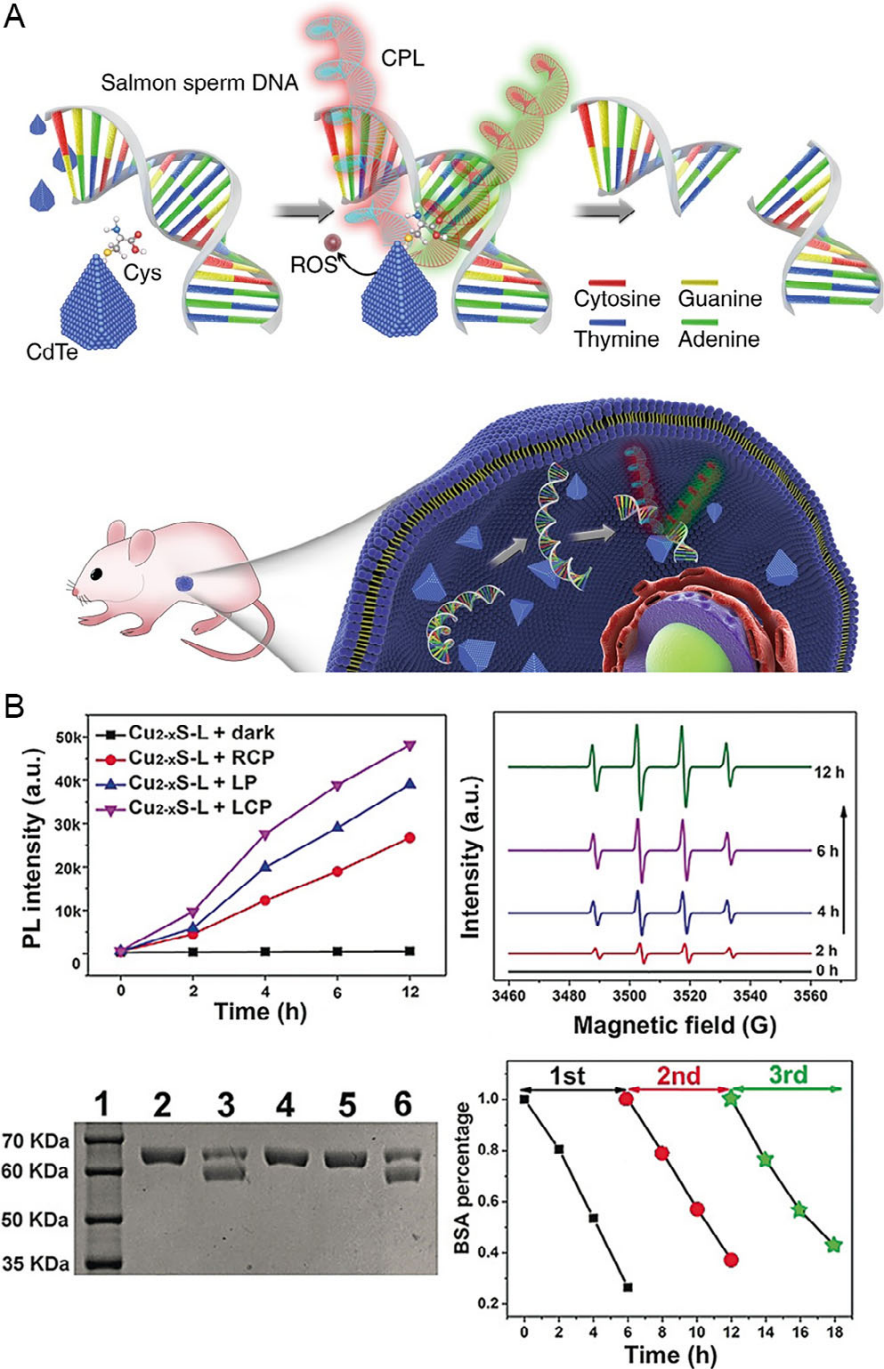
A) DNA cleavage achieved by ROS generated by chiral CdTe nanoparticles. Reproduced with permission.^[^ ^57^ ^]^ Copyright 2018, Springer Nature. B) Chiral QDs produce ·OH to cleave BSA. Reproduced with permission.^[^ ^37^ ^]^ Copyright 2019, Wiley‐VCH.

Natural enzymes, whose main components are proteins, are capable of efficiently catalyzing replicative chemical reactions such as metabolism and energy conversion in living organisms and are widely used in industry.^[^ ^53^ ^]^ However, instability and expensive costs limit the development of natural enzymes. In recent years, low‐cost and stable nanoenzymes such as Fe_3_O_4_ nanoparticles have been developed^[^ ^54^ ^]^ and have achieved good results in biosensing,^[^ ^55^ ^]^ disease diagnosis, and treatment.^[^ ^56^ ^]^ By regulating the production of ROS, chiral nanomaterials achieve enzymatic effects in living organisms. Chiral cysteine‐modified CdTe nanoparticles can generate ROS under 405 nm laser irradiation. This unique property allows these chiral nanoparticles to cleave a specific DNA sequence site.^[^ ^57^ ^]^ When mixed with chiral CdTe nanoparticles and subjected to laser irradiation, DNAs that are over 90 bp in size and containing a GAT’ATC sequence are cleaved into multiple bands (**Figure** **5**A). Following this reaction, these nanoparticles form nanorods. This specific DNA cleavage process involves the generation of hydroxyl radicals as the sole ROS. The high affinity of this chiral nanoparticles for the GAT’ATC sequence is crucial for selective cleavage, as mutated DNA lacking this sequence does not undergo the same reaction. The entire cleavage process produces only one hydroxyl radical. Moreover, chiral nanomaterials can also catalyze the cleavage of proteins.^[^ ^37^ ^]^ The chiral penicillamine molecule‐modified Cu_2−*x*_S QDs generated many hydroxyl radicals under circularly polarized light irradiation to achieve the catalysis of bovine serum albumin (BSA) (Figure 5B). The improved cleavage effect of L‐QDs is due to their higher affinity with BSA. The key role of penicillamine is clearly evident as other chiral molecules do not produce the same cleavage effect on BSA. These examples showcase how chiral nanomaterials can be designed to replicate the catalytic effects of natural enzymes, selectively targeting specific biomolecules and enabling controlled reactions using light irradiation. The development of stable and cost‐effective nanoenzymes opens up new possibilities for various biomedical and industrial applications.

## 8 Microbial Inhibition

**Figure 6:**
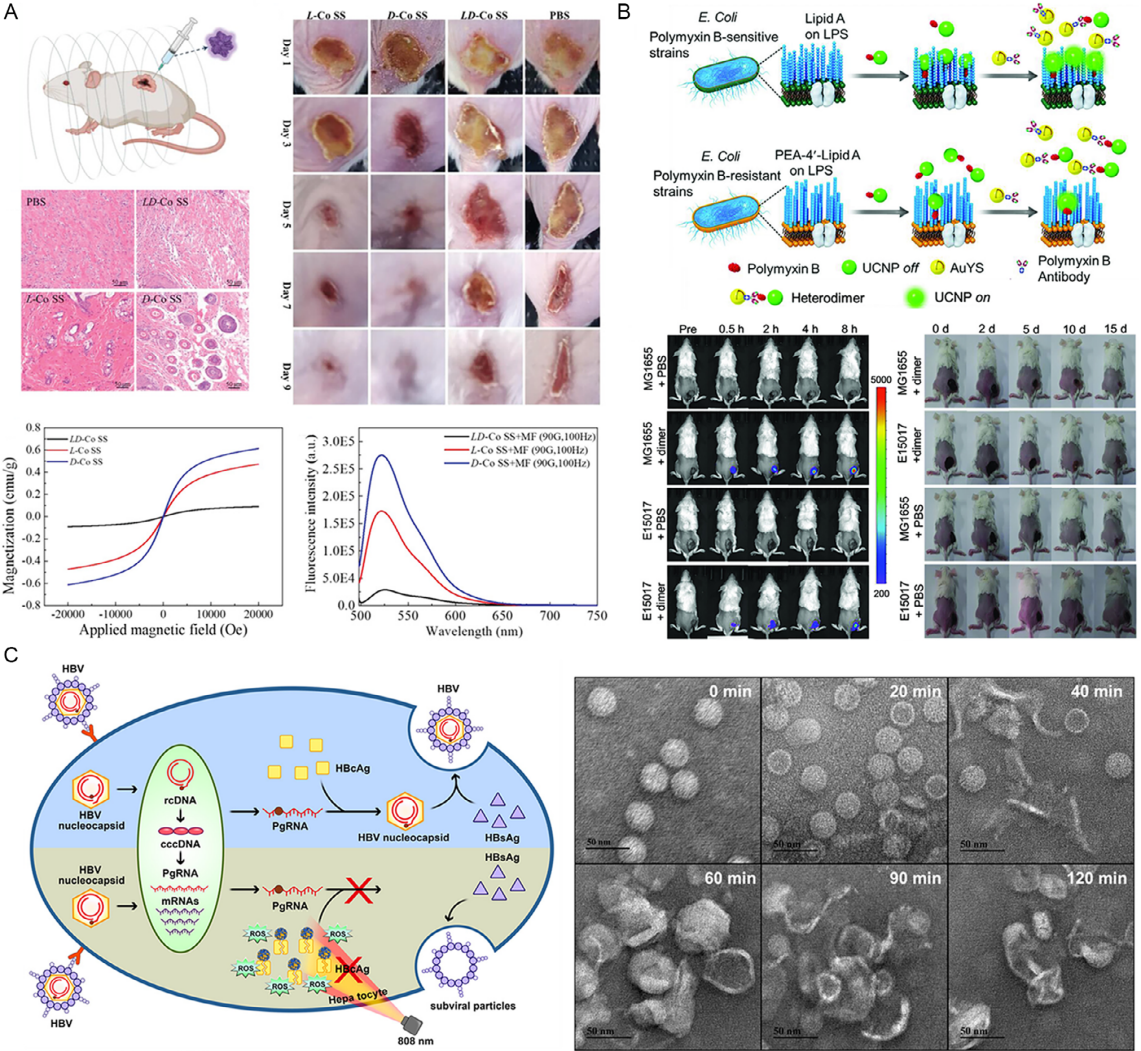
A) Chiral Co SS combines with electromagnetic fields to produce singlet oxygen against *Staphylococcus aureus*. Reproduced with permission.^[^ ^38^ ^]^ Copyright 2022, Wiley‐VCH. B) Au YS–UCNP heterodimer enables imaging and treatment of inflammation sites in mice. Reproduced with permission.^[^ ^61^ ^]^ Copyright 2018, Wiley‐VCH. C) Chiral copper sulfide nanoparticles produce large amounts of ROS that inhibit HBV assembly. Reproduced with permission.^[^ ^63^ ^]^ Copyright 2021, Wiley‐VCH.

Due to drug abuse and the continuous deterioration of the environment, pathogenic microorganisms have become a serious threat to public health.^[^ ^58^ ^]^ The development of traditional medicines requires significant cost and time. Therefore, there is an urgent need for new interventional methods for pathogenic microorganisms. Over recent years, researchers have developed new inorganic nanomaterials for the inhibition of pathogenic microorganisms and achieved good results.^[^ ^59^ ^]^ However, most broad‐spectrum antimicrobial nanomaterials are nonselective and toxic; these represent significant limitations.^[^ ^60^ ^]^ Chiral inorganic nanomaterials can address this issue in a highly effective manner. For example, Wang et al. adjusted the concentration of tartaric acid to prepare a series of chiral cobalt superstructures (Co‐SS) with different morphologies.^[^ ^38^ ^]^ Of the five chiral cobalt superstructures, a chiral hexagram structure could generate ROS to kill Gram‐positive *Staphylococcus aureus* under the action of an electromagnetic field (**Figure** **6**A). Due to the chirality‐induced spin‐selectivity effect, D*‐*Co‐SS produced a 1.59‐fold higher active oxygen content than L*‐*Co‐SS, thus resulting in stronger bactericidal ability. In addition, heterodimers formed by UCNPs and gold yolk‐shell nanoparticles achieved dual‐signal detection of polymyxin‐B‐resistant *Escherichia coli* and also generated ROS, thus leading to bacterial elimination under 980 nm illumination, thus treating inflammation.^[^ ^61^ ^]^ Compared with previous detection methods, chiral dimers significantly shorten the detection time for drug‐resistant bacteria, thus providing a new strategy for the rapid detection of drug‐resistant bacteria in the clinic (Figure 6B). The advantages of chiral nanomaterials, such as their unique structure, small size, and surface chemical properties, make them effective antiviral tools. Ultrasmall chiral NPs are highly active due to their relatively large surface‐to‐volume ratio.^[^ ^62^ ^]^ Tripeptide‐modified chiral Cu_2_S NPs can bind specifically to HBcAg and produce a large amount of hydroxyl radicals, singlet oxygen, superoxide anions, and other ROS after 808 nm light irradiation, thus resulting in the cleavage of HBcAg.^[^ ^63^ ^]^ The cleavage activity of L‐Cu_2_S NPs was stronger as it produced more ROS. Chiral Cu_2_S nanoparticles were shown to successfully cure hepatitis B virus (HBV) in mice (Figure 6C).

## 9 Neurodegenerative Diseases

**Figure 7:**
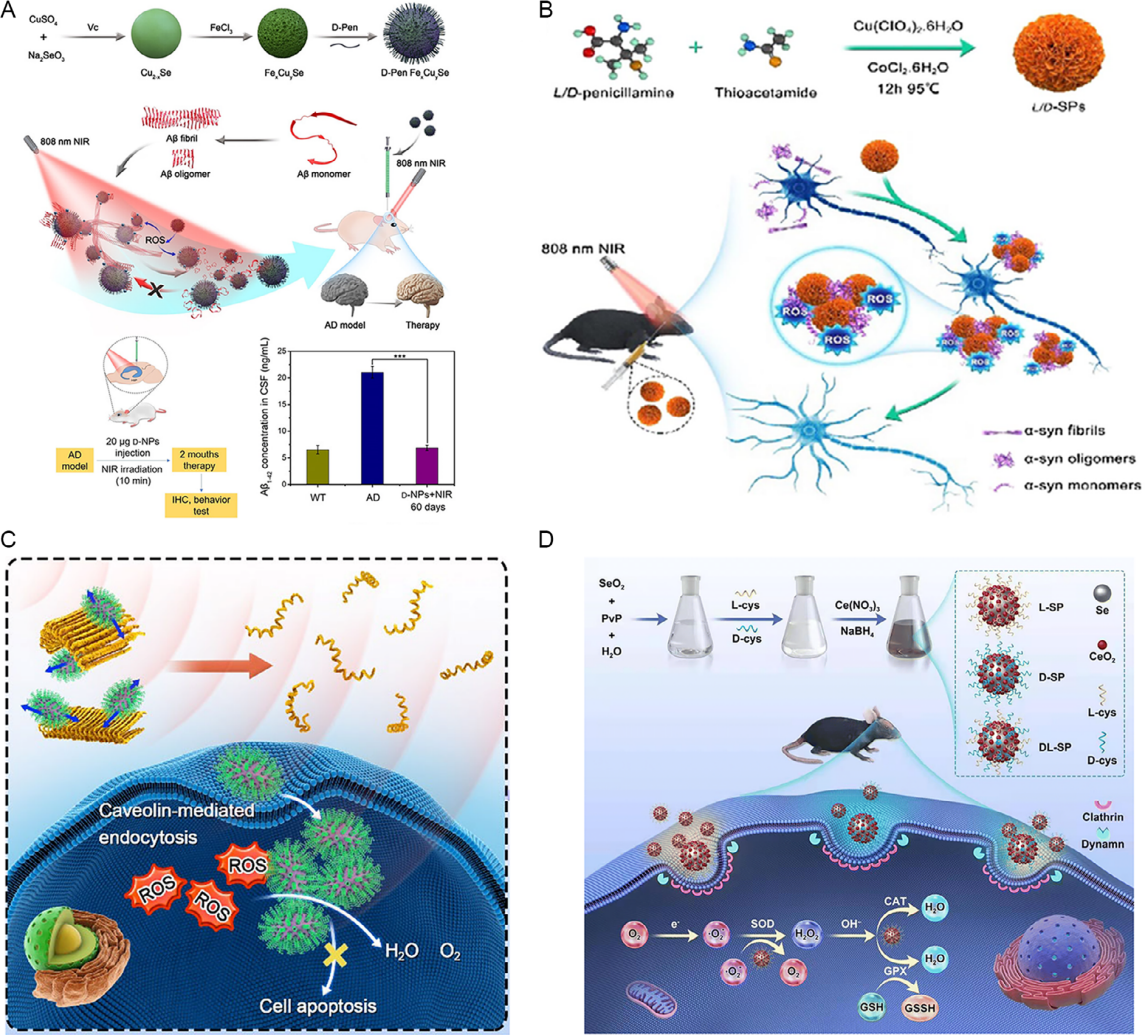
A) Chiral L/D‐Fe_*x*_Cu_*y*_Se nanoparticles produce ROS to degrade β‐amyloid plaques in the brain. Reproduced with permission.^[^ ^66^ ^]^ Copyright 2020, Wiley‐VCH. B) NIR light induces chiral SPs clear α‐syn to relieve symptoms of Parkinson's disease in mice. Reproduced with permission.^[^ ^67^ ^]^ Copyright 2022, Chinese Chemical Society. C) Chiral Mn2O3 NPs combined with magnetic field synergistically cleared excess ROS and α‐syn in mice. Reproduced with permission.^[^ ^68^ ^]^ Copyright 2022, Springer. D) Multienzyme active chiral Se@CeO2 SPs alleviated neurodegeneration in mice. Reproduced with permission.^[^ ^69^ ^]^ Copyright 2023, Royal Society of Chemistry.

Neurodegenerative diseases, such as Alzheimer's disease (AD) and Parkinson's disease (PD), are experiencing a global increase in morbidity and mortality.^[^ ^64^ ^]^ An expanding body of reliable studies has demonstrated the molecular mechanism by which the excessive production of ROS can promote the generation of neurodegenerative diseases.^[^ ^65^ ^]^ Chiral Fe_x_Cu_y_Se nanoparticles have been shown to generate ROS under 808 nm NIR light, thus leading to the disintegration of Aβ protofibrils and a reduction in the aggregation of Aβ monomers.^[^ ^66^ ^]^ ITC results revealed that D‐Fe_*x*_Cu_*y*_Se preferentially binds to Aβ proteins, thus explaining the enhanced scavenging efficiency of D‐NPs when compared to L‐NPs. ESR spectroscopy further revealed that D‐NPs produced both hydroxyl radicals and singlet oxygen as ROS under NIR light irradiation, thus causing a change in the conformation of β‐sheets. D‐NPs were shown to effectively improve the cognitive and memory abilities of AD mice (**Figure** **7**A). Similarly, chiral Cu_x_Co_y_S superparticles were shown to be capable of modulating ROS to achieve α‐synuclein catabolism under 808 nm light; moreover, D‐SPs produced 1.42‐fold more ROS than L‐SPs.^[^ ^67^ ^]^ As the symptoms of PD mice were alleviated, the D‐SPs gradually decomposed into smaller nanoparticles and were excreted through the blood–brain barrier (Figure 7B). Nanoenzymes with ROS scavenging ability have been widely investigated and provide a promising solution to the disadvantages of natural enzymes associated with preparation and poor stability. Researchers found that chiral Mn_2_O_3_ NPs synthesized with tartaric acid as the ligand exhibited ROS scavenging functionality.^[^ ^68^ ^]^ As a nanoenzyme, D‐NPs exhibit higher efficiency for scavenging intracellular ROS levels and provide better protection for neuronal cells when compared to L‐NPs, due to their higher cellular uptake capacity (Figure 7C). In addition, the introduction of a noninvasive magnetic field generated mechanical forces that promoted the inhibition of α‐synuclein fibrosis by chiral nanoparticles. The cysteine‐modified chiral Se@CeO_2_ superparticles possessed multienzyme activity.^[^ ^69^ ^]^ Chiral Se@CeO_2_ demonstrated higher catalytic activity when compared to selenium nanoparticles and cerium dioxide nanoparticles; furthermore, L‐SPs exhibited stronger resistance to oxidative stress‐induced cell damage and could improve neurodegenerative lesions in PD mice; this was attributed to the selectivity of cells for chiral nanomaterials (Figure 7D). The combination of chiral recognition and applied field induction provides a strategy for nanoparticles to remove the build‐up of abnormal proteins, thereby alleviating neurodegenerative diseases.

## 10 Tumor Therapies

Cancer stands as the foremost cause of human health‐related mortality. Currently, the clinical treatment of cancer involves surgical resection and chemotherapy. However, traditional cancer treatment methods suffer from significant disadvantages, such as poor targeting, a high susceptibility to recurrence, and metastasis. To overcome these limitations, chiral nanomaterials have been developed for tumor therapy, thus leading to significant achievements.^[^ ^8^, ^70^ ^]^ Chiral nanomaterials possess a strong ability to absorb circularly polarized light, thus making photodynamic therapy the main approach for using chiral nanomaterials in tumor treatment.

**Figure 8:**
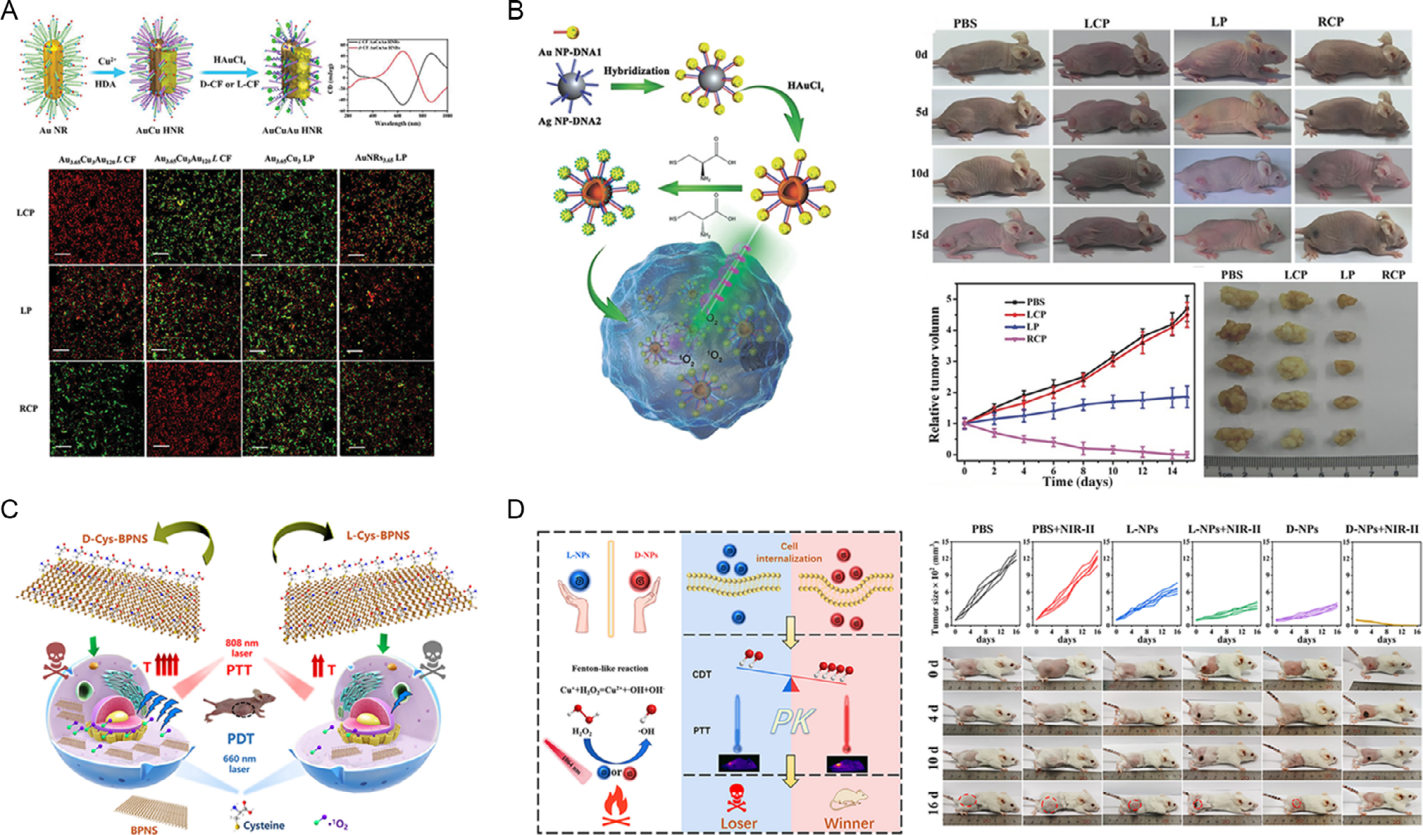
A) Photodynamic therapy of tumors by chiral plasmonic AuCuAu heteronanorods. Reproduced with permission.^[^ ^71^ ^]^ Copyright 2020, Wiley‐VCH. B) Cysteine‐modified chiral SS nanostructures can be used as photosensitizers. Reproduced with permission.^[^ ^46^ ^]^ Copyright 2017, Wiley‐VCH. C) The photodynamic therapeutic effect of chiral BPNS showed a hands‐dependent effect. Reproduced with permission.^[^ ^73^ ^]^ Copyright 2023, American Chemical Society. D) Chiral Cu_2−*x*_Se nanoparticles’ increase apoptosis of cancer cells by generating hydroxyl radicals through Fenton reaction. Reproduced with permission.^[^ ^74^ ^]^ Copyright 2021, American Chemical Society.

The chiral AuCuAu heterostructure, synthesized using a two‐step method with CF dipeptide as a ligand, exhibits robust chiral optical activity.^[^ ^71^ ^]^ This chiral heterojunction effectively absorbs enantiomeric circularly polarized light, thus providing photothermal and photodynamic therapy to Hela cells (**Figure** **8**A). After 15 days of tail vein treatment, the tumors in experimental mice completely disappeared. Chiral SSs gold nanostructures (CAM), propelled by cysteine‐modified DNA, demonstrated the ability to generate a substantial amount of ROS and exhibit excellent dispersibility in cells, making these a promising chiral photosensitizer for a wide range of applications.^[^ ^46^ ^]^ Both in vitro and in vivo experiments have revealed the outstanding antitumor ability of chiral SS structures, with D‐Cys‐SS15 exhibiting the most effective photodynamic treatment effects under circularly polarized light irradiation, thus leading to the complete eradication of tumor tissue in mice after 15 days of injection (Figure 8B). As a common nanodrug, black phosphorus nanosheets are not readily retained in tumor cells,^[^ ^72^ ^]^ thus leading to a significant reduction in their therapeutic effect. However, chiral BPNS modified with cysteine can prolong their intracellular residence time, thereby enhancing their photothermal and photodynamic therapy efficacy on tumors.^[^ ^73^ ^]^ D‐Cys‐BPNS exhibits higher cellular uptake and resistance to catabolism, thus resulting in enhanced ROS generation and stronger antitumor activity. The modification of chiral ligands offers a strategy to improve the stability and therapeutic efficacy of BPNS (Figure 8C). By functioning as a Fenton catalyst, penicillamine‐modified chiral Cu nanoparticles can catalyze the generation of hydroxyl radicals from hydrogen peroxide in tumor cells for CDT.^[^ ^74^ ^]^ In vivo experiments have demonstrated that D‐NPs, under NIR light irradiation, exhibit stronger photothermal effects and ROS generation, thus leading to complete tumor elimination, while L‐NPs do not; these effects are related to the selective cellular uptake of nanomaterials (Figure 8D).

## 11 Other Diseases

**Figure 9:**
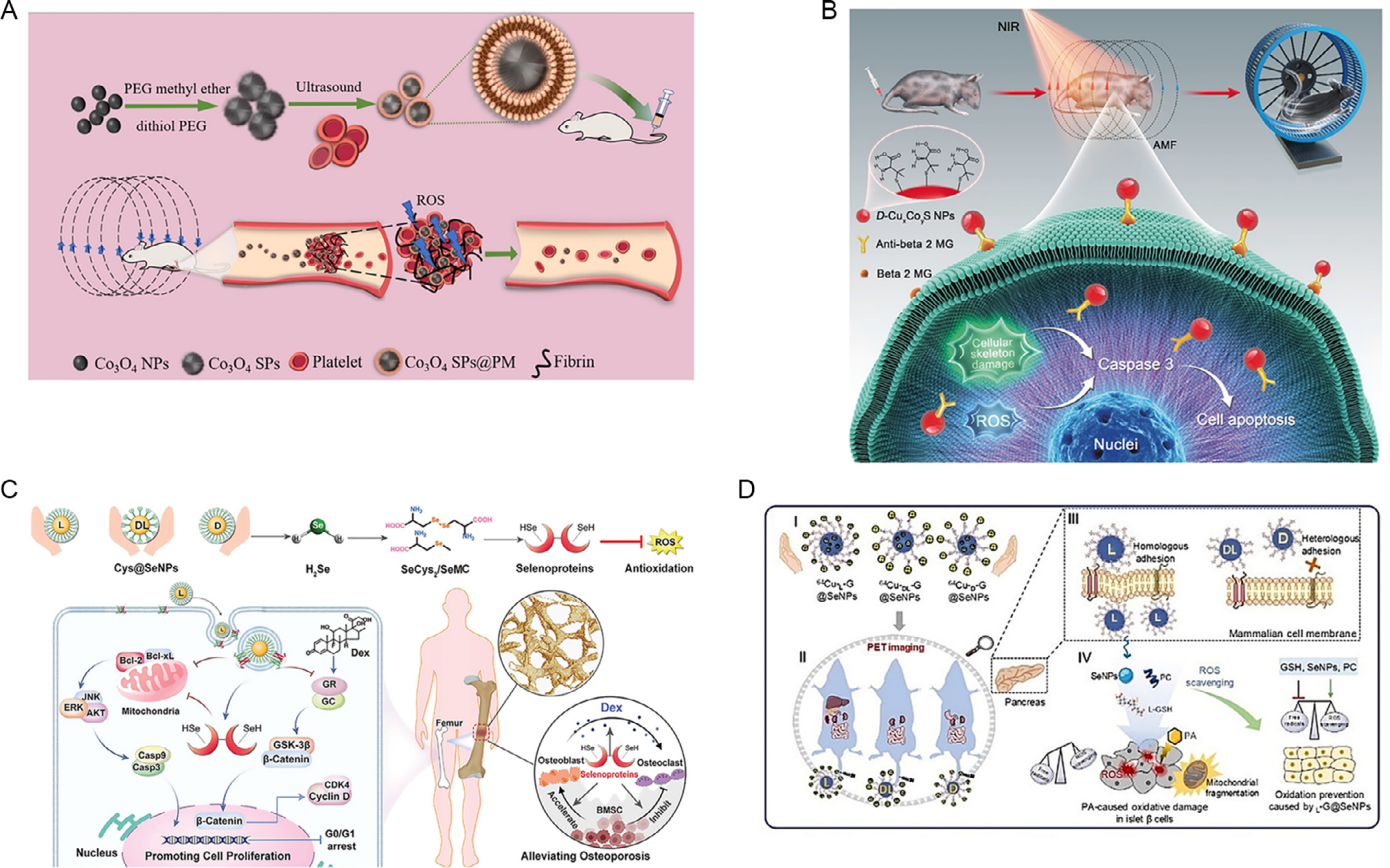
A) Chiral Co_3_O_4_ generates ROS to lyse the thrombi. Reproduced with permission.^[^ ^78^ ^]^ Copyright 2021, Wiley‐VCH. B) Elimination of senescent cells with AMF and NIR illumination using chiral Cu_*x*_Co_*y*_S NPs. Reproduced with permission.^[^ ^79^ ^]^ Copyright 2020, Wiley‐VCH. C) Treatment of osteoporosis by removal of excessive ROS by cysteine‐modified chiral selenium nanoparticles. Reproduced with permission.^[^ ^81^ ^]^ Copyright 2023, Wiley‐VCH. D) L‐G@SeNPs can prevent palmitic acid‐induced antioxidant. Reproduced with permission.^[^ ^82^ ^]^ Copyright 2023, Wiley‐VCH.

By regulating ROS, nanomaterials hold the potential to treat various other diseases, including inflammation,^[^ ^75^ ^]^ along with cardiovascular and cerebrovascular diseases.^[^ ^76^ ^]^ Venous thromboembolism (VTE) features blood clots that are rich in fibrin and red blood cells (RBCs). Existing antithrombotic drugs can achieve therapeutic effects by activating proteases to dissolve fibrin, but they often lead to side effects such as bleeding and the lack of precise targeting.^[^ ^77^ ^]^ To address these issues, chiral Co_3_O_4_ superparticles with paramagnetic properties have been employed in thrombus therapy.^[^ ^78^ ^]^ Wang et al. coated the SPs with a layer of platelet membrane to facilitate targeting of the thrombus site (**Figure** **9**A). Chiral Co3O4SPs@PM were shown to specifically target the thrombus site in experimental mice and generate a substantial amount of singlet oxygen in situ to degrade fibrin and dissolve blood clots. Under the influence of an electromagnetic field, D‐SPs exhibited higher thrombolytic efficiency than L‐SPs; furthermore, the survival rate of a mouse model of hyperthrombosis treated with D‐SPs for 25 days reached 70%. The chirality‐induced spin selectivity effect resulted in a greater generation of active oxygen from D‐SPs. In addition, utilizing a photomagnetic effect, chiral Cu_x_Co_y_S NPs selectively induced the apoptosis of senescent cells under near‐infrared irradiation and alternating magnetic field (AMF) exposure.^[^ ^79^ ^]^ The high expression of Beta 2 MG on senescent cells prompted the modification of chiral nanomaterials with Beta 2 MG antibodies to enhance their selectivity and minimize damage to normal cells during AMF treatment (Figure 9B). The cellular uptake efficiency of D‐Cu_*x*_Co_*y*_S NPs into senescent cells was 2.5‐fold higher than that of L‐Cu_*x*_Co_*y*_S NPs, thus suggesting better membrane component binding of D‐NPs. The synergistic effect of light and AMF led to significantly higher ROS generation and cytoskeletal damage in aging cells than single‐condition treatments, thus resulting in the increased expression of caspase 3, a key protease involved in apoptosis.

Furthermore, based on the link between the trace element selenium and bone formation,^[^ ^80^ ^]^ Chen et al. designed cysteine‐modified chiral SeNPs for the treatment of osteoporosis.^[^ ^81^ ^]^ Chiral selenium nanoparticles were able to scavenge excessive ROS in DEX‐induced osteoblasts and alleviate osteoporosis symptoms (Figure 9C). The mechanism involved chiral selenium nanoparticles activating the WNT pathway after entering the cell and upregulating the level of antioxidant selenoproteins. The ROS scavenging ability of L‐Cys@SeNP is stronger than that of D‐Cys@SeNP; this was attributed to the chiral selectivity of biomolecules affecting their distribution and transport. Moreover, GSH‐modified Se NPs were shown to mitigate the oxidative damage caused by palmitic acid in insulinoma cells. Palmitic acid‐induced apoptosis often leads to mitochondrial fragmentation and elevated ROS production.^[^ ^82^ ^]^ GSH@SeNPs treatment also prevented mitochondrial fragmentation in ins‐1e cells, reduced ROS production against oxidative stress, and L‐type nanoparticles exhibited a more pronounced effect than the D‐type, possibly due to the stronger homologous adhesion of L‐GSH to the cell membrane of INS‐1 E cells, thus resulting in higher cellular uptake of L‐SeNPs (Figure 9D).

## 12 Conclusion and Future Perspectives

**Figure 10:**
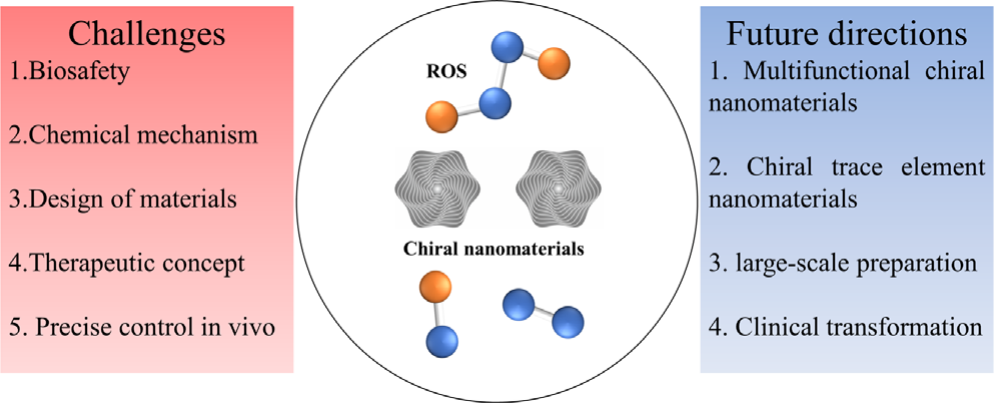
The challenges and future directions of ROS‐associated chiral nanomaterials.

The modulation of ROS using chiral nanomaterials presents both promising opportunities and challenges in various biomedical applications (**Figure** **10**). ROS modulation is a delicate process, as ROS acts as a double‐edged sword, exerting both beneficial and harmful effects. Therefore, the design of chiral nanomaterials should focus on responsiveness to the microenvironment to ensure the accurate monitoring and regulation of ROS concentration and distribution in pathological regions. One critical aspect is achieving precise control over ROS generation and degradation pathways in vivo. Chiral nanomaterials must be engineered to achieve the desired therapeutic effect while minimizing harm to normal cells surrounding the target site. Understanding the molecular mechanisms underlying the biological effects of chiral nanomaterials is essential if we are to optimize their design and ensure their safe and effective use. Future applications of chiral nanomaterials in ROS modulation hold great promise in various fields. By tuning the physical and chemical properties of chiral nanomaterials, targeted ROS generation can be achieved in specific cell types, tissues, or organs. Multifunctional chiral nanomaterials integrating fluorescent probes and drug carriers can serve as versatile platforms for diagnosis, therapy, and monitoring. Personalized medicine can also benefit from chiral nanomaterials as they can be designed and tailored to fit individual patient needs based on personalized assessments. Harnessing the unique bioactivity of trace elements, the development of chiral trace element nanomaterials opens new avenues for biomedical applications. In terms of large‐scale production, chiral nanomaterials offer simplicity compared to other inorganic nanomedicines, making them more accessible for clinical use. Collaboration between universities, hospitals, and enterprises will be crucial to facilitate the translation of chiral nanomaterials from research to clinical applications.

Overall, the future of chiral nanomaterials in ROS modulation holds immense potential for disease treatment and other biological applications. As scientific research and technological advancements continue, chiral nanomaterials are likely to play an increasingly significant role in the medical and life science fields, leading to new opportunities and innovations.

## Conflict of Interest

The authors declare no conflict of interest.
